# Dietary pyrroloquinoline quinone improvement of the antioxidant capacity of laying hens and eggs are linked to the alteration of Nrf2/HO-1 pathway and gut microbiota

**DOI:** 10.1016/j.fochx.2023.101021

**Published:** 2023-11-30

**Authors:** Dan Shao, Liangji Liu, Haibing Tong, Shourong Shi

**Affiliations:** Poultry Institute, Chinese Academy of Agricultural Sciences, Yangzhou 225125, China

**Keywords:** Pyrroloquinoline quinone disodium, Egg quality, Antioxidant capacity, Gut microbiota, Antioxidant enzymes

## Abstract

Pyrroloquinoline quinone disodium (PQQ·Na_2_) has been considered a human food supplement for human health promotion with its antioxidant properties. To determine whether PQQ·Na_2_ had similar functions to improve the antioxidant ability of layers and eggs, 180 laying hens were fed with 0 or 0.4 mg/kg PQQ·Na_2_ diets. Supplementation with PQQ·Na_2_ increased the albumen height, Haugh unit of the eggs. PQQ·Na_2_ also led to a higher glutathione peroxidase (GSH-Px) concentration in plasma and a lower malondialdehyde (MDA) content in the liver and egg yolk. Similarly, liver gene and protein expression of nuclear factor erythroid 2-related 2 (Nrf2) and heme oxygenase 1 (HO-1) were up-regulated by PQQ·Na_2._ Moreover, PQQ·Na_2_ increased the abundance of *Firmicutes*, *Microbacterium, Erysipelatoclostridium, Mailhella, Lachnospiraceae_UCG-010,* and *Herbaspirillum* in gut. Overall, these results suggested PQQ·Na_2_ increased the antioxidant ability of layers and eggs which might be in connection with the activation of the Nrf2/HO-1 pathway and optimized gut microflora.

## Highlights

• The effect of PQQ on antioxidant capacity of laying hens and eggs is investigated.
• PQQ can positively activate the Nrf2/HO-1 pathway to improve hen and egg antioxidative status.
• PQQ improves antioxidant capacity of laying hens and eggs by increasing beneficial gut microbiota.

## 1 Introduction

Today, foods are not only used to offer essential nutrients for humans, but also applied to stem nutrition-related diseases and further consumers’ well-being (Siro et al., 2008). Eggs play a considerable part in basic nutrition for humans, particularly for some special populations for example the aged, children, gestational women, and people who are in sports training and convalescents (Miranda et al., 2015). As a cheap and highly nutritious cost-effective food, eggs compound with an immunomodulator, antimicrobial, anti-cancer, antioxidant, and anti-hypertensive properties (Abeyrathne,Lee,& Ahn, 2013). Particularly, eggs are regarded as a great source of dietary antioxidants (Nimalaratne & Wu, 2015), which obtains major interest from researchers owing to their protection of humans from degenerative processes (Natoli et al., 2007). And scientific evidence has shown additives with antioxidant function could improve the antioxidant capacity of eggs (Wang et al., 2020, Wang et al., 2020).

In recent years, an increasing attraction in the study of the antioxidant effects of pyrroloquinoline quinone (4,5-dihydro-4,5-dioxo-1H-pyrrolo[2,3-f] quinolone-2,7,9-tricarboxylic acid, PQQ) has been considered. PQQ is a water-soluble thermostable triglyceride-quinone (Zhang, Feustel, & Kimelberg, 2006), and the free radical-scavenging activity of PQQ is 7.4 times higher than that of vitamin C, and it has been considered the most effective water-soluble antioxidant (Akagawa, Nakano, & Ikemoto, 2016). As a novel redox cofactor, PQQ has been reported to enhance growth and stress tolerance (Misra, Rajpurohit, & Khairnar, 2012), and is widely considered a food additive for health improvement and disease prevention (Geng et al., 2019). On account of its utility functions (Akagawa, Nakano, & Ikemoto, 2016), PQQ disodium (PQQ·Na_2_) salt has been confirmed as a natural health product by Natural Health Products Directorate in Canada (Samuel et al., 2015), meanwhile, PQQ·Na_2_ has also been categorized as a new type of food in Japan in 2013 and defined as a dietary supplement in European in 2018 (Wang et al., 2020).

The functional capabilities of PQQ have also been applied to animal nutrition. Diet administration of PQQ was found to prevent microbial imbalance and ease liver fibrosis of obese mice (Friedman et al., 2018). Diet supplementation of PQQ was shown to alleviate jejunal mucosal inflammatory injury and regulate the dysbiosis of gut microbiota in piglets infected with *E. coli* K88 (Huang et al., 2020). Besides, PQQ-supplemented diets were observed to preserve the livers against damage of weaned pigs via the upregulated the relative gene and protein expression of Nrf2, HO-1 in the Nrf2/HO-1 pathway (Huang et al., 2021), a signaling pathway that played a protective role in oxidative stress (Zhao et al., 2019, Zhao et al., 2019). Moreover, PQQ·Na_2_ was considered a possibly practical feed additive for ameliorating feed efficiency, antioxidant ability and meat quality of broilers (Wang et al., 2015). The protective effects of PQQ·Na_2_ against liver damage were observed which might be partially due to the enhancement of antioxidant defense systems of laying hens (Wang et al., 2016). In our preliminary study, laying hens fed PQQ·Na_2_-supplemented diets (0.1, 0.2, 0.4, 0.8, 1.6, 3.2 mg/kg) showed that diet with 0.4 mg/kg PQQ·Na_2_ had better laying performance and antioxidant capacity of eggs (unpublished data). However, there is little information concerning how dietary PQQ·Na_2_ supplementation of hens affects the quality and antioxidant capacity of eggs.

Hence, the present study is carried out to discuss the reply of laying hens to dietary PQQ·Na_2_ supplementation by assessing antioxidant capacity of the hens and eggs, expression level of Nrf2/HO-1 signaling pathway and caecum microflora status.

## 2 Materials and methods

### 2.1 Chemicals and reagents

PQQ·Na_2_ was acquired from Haotian Pharmaceutical Co. Ltd (Shandong, China), with a purity of >98 %. The antibodies of Nrf2 and HO-1were purchased from Proteintech Group, Inc (United States), and the antibody of β-actin was purchased from Biosynthesis Biotechnology Co. Ltd (Beijing, China).

### 2.2 Animals and management

All animal procedures were evaluated and agreed by the Animal Care and Use Committee, Poultry Institute, Chinese Academy of Agriculture Science, under permit NO.2022–0035 (Jiangsu, China). 180 (37-week-old) Hy-line laying hens with alike egg production performance were stochastically chosen and split into 2 groups with 6 replicates of 15 hens (1 hen per cage, 23 cm width × 38 cm length × 39 cm height). After a 1-week adaptation with basal diet, 2 groups included a basal diet group (CON) and a basal diet supplemented with 0.4 mg/kg PQQ·Na_2_ (PQQ) for 8 weeks (the dose was based on the previous study (Wang et al., 2015) and our study (unpublished data)). The basal diet (Table S1) was programmed to conform to the requirement of laying hens of NRC. All hens were raised in a ventilated room with a mean temperature of 21 ± 2 ℃, a relative humidity of 65 ± 3 % and a photoperiod of 16 h daily light. Water and feed were available ad libitum throughout the complete experiment.

### 2.3 Laying performance

The number and weight of eggs were documented every day. The amount of provided feed and residual feed were recorded weekly. Feed intake (g/hen per d), egg production (%), and average egg weight (g/hen per d) by each replicate were recorded weekly. The feed conversion ratio (FCR) was computed as the total feed consumption divided by the total egg weight (feed/egg, g/g). Daily egg mass was calculated as average egg weight × egg production (g/hen per d).

### 2.4 Egg quality

At week 8, egg samples (5 eggs per replicate) were randomly converged to evaluate the egg quality traits. The yolk weight was individually weighed. The eggshell strength was detected via an eggshell thickness gauge (EFR-01, ORKA Food Technology, Ltd., Ramat Hasharon, Israel), and the egg weight, yolk color, albumen height, and the Haugh unit (HU) were analyzed via an Egg Multi Tester (EA-01, ORKA Food Technology, Ltd., Ramat Hasharon, Israel). A colorimeter QCR-SC (TSS Company, Britain) was used to analyze the eggshell color value. A shell thickness gauge (211-101F, Guanglu Measuring Instrument Co., Ltd., Guilin, China) was used to determine the eggshell thickness. The thickness was calculated with the average value of the bottom, middle, and top of the eggshell thickness (Zhao et al., 2021).

### 2.5 Assessment of the antioxidant capacity and freshness of eggs

Egg samples (25 eggs per replicate) were gathered by the end of week 8 of the supplementation period for 3 days and then store in the egg repository (temperature 18℃, humidity 75 %) until the measurement period was over. Egg weight, Egg air diameter, egg albumen pH value, albumen height, HU, and yolk malondialdehyde (MDA) were analyzed with 5 eggs per replicate every 7 days. MDA concentrations were detected via commercial kits. Egg air diameter was measured with vernier caliper determination (B26976, Guanglu Measuring Instrument Co., Ltd., Guilin, China) (Dong et al., 2018). Egg albumen pH value was tested using a laboratory pH meter (PHM 220, Radiometer, Copenhagen).

### 2.6 Plasma, liver antioxidant enzyme activity assay

At week 8, 1 bird was casually chosen from each replicate. 2 mL blood sample was obtained from the left wing vein and stored in a 5 mL EDTA sodium anticoagulant tube. After centrifugation (3500 r/min, 10 min, 4℃), plasma was obtained for antioxidant indices analysis. After that, birds were slaughtered by cervical dislocation for liver samples and cecal contents collection. The liver samples were used for antioxidant indices analysis, as well as real-time PCR and Western blot analysis. The cecal contents were applied to 16S rRNA sequencing.

The activity of the malondialdehyde (MDA), glutathione peroxidase (GSH-Px), superoxide dismutase (SOD), and catalase (CAT) in plasma and liver were detected by the commercial kits (A003-1–2, A001-1–1, A005-1–2, A007-1–1, respectively). All kits were conducted in accordance with the manufacturer’s instructions. The absorbance of the four antioxidant indices were measured with a microplate reader (Infinite M200 Pro, Tecan, Switzerland).

### 2.7 RNA extraction and real-time PCR

Total RNA from liver samples were extracted with TRIzol reagent kit. Then cDNA was reverse transcribed by a reagent kit. The real-time PCR was carried out with SuperReal PreMix Plus, and conducted in an ABI 7500 Step One Plus instrument (Applied Biosystems, Foster City, CA, United States). The primer sequences were shown in Table S2. The relative quantification of mRNA expression of the genes were determined by the method of 2^-△△Ct^ (Pfaffl et al., 2002).

### 2.8 Western blot analysis

The total protein contents were draw from livers using RIPA Lysis Buffer and their concentrations were analyzed with BCA Protein Assay kit. We denatured each protein sample for 5 min at 100 ℃ before loading it into polyacrylamide gel, and then transfered to polyvinylidene fluoride membranes. After that, we washed the membranes in Tween-Tris-buffered saline buffer and blocked it for 2 h, and then cultivated it with primary antibodies overnight at 4 ℃. The primary antibodies were listed as bellows: Nrf2 (16396–1-AP, 1:1000), HO-1 (10701–1-AP, 1:1000), β-actin (BS-0061R, 1:1000). After washes, the membranes were incubated with anti-rabbit IgG secondary antibody for 2 h. Then the immunoblots were visualized with Immobilon® Western Chemiluminescent HRP Substrate (WBKLS0500, Millipore Corporation, United States) and quantified via the Bio-Rad Chemidoc^TM^ XRS + (Bio-Rad, United States). The band densities of proteins were normalized with those of β-actin.

### 2.9 Cecal content flora structure analysis

Microbial DNA was extracted from each bird’s cecal content with the EZNA Soil DNA kit (D5625-02). The concentration and purity of microbial DNA were detected with a NanoDrop ND-2000 spectrophotometer (NanoDrop Technologies, Wilmington, DE) and store at −20 °C for further analysis. The V3/V4 region of 16S rRNA was PCR-amplified via polymerase chain reaction. The amplified products were extracted from 2 % agarose gels and purified with the Qiagen Gel Extraction Kit (Qiagen, Germany). Libraries were then prepared with NEBNext® Ultra™ IIDNA Library Prep Kit (Cat No. E7645) and their quality was evaluated on the Qubit@ 2.0 Fluorometer. Finally, the library sequencing with the Illumina Novaseq platform and biology analysis were performed in Novogene Bioinformatics Technology Co., Ltd (Beijing, China).

The original data of each sample was split based on the barcode, and then spliced by FLASH V 1.2.11. After that, high-quality sequences were obtained using the faster (Version 0.20.0) software and clustered using Uparse software V 7.0.1001. Then the sequences were gathered into Amplicon Sequence Variants (ASVs) with 100 % identity. At the meantime, the most confirmed ASVs sequences were chosen as the representative ASVs sequences, and were annotated. After uniformizing the sample data, the sample componential analysis (Alpha Diversity) and multi-sample comparative analysis (Beta Diversity) were computed to break down the diversity of samples based on the ASVs levels. At the same time, the R software (Version 3.5.3) was used to do T-test analysis and the LEfSe software (Version 1.0) was applied to make LEfSe analysis (LDA score threshold: 3) to run down the significantly different species.

### 2.10 Statistical analysis

Each replicate was done duty for an experimental unit. The whole statistical analyses were performed by using the *t*-test in SPSS 26.0 software (SPSS Inc., Chicago, United States), and all the experimental results were plotted with the mean value ± standard error (SE), and difference was declared at *P* < 0.05 and difference tendency was recognized as significant at 0.05 < *P* < 0.1. The histograms and line charts were developed using GraphPad Prism 9 software (GraphPad Prism Inc., USA).

## 3 Results

### 3.1 Production performance and egg quality

Dietary PQQ·Na_2_ supplementation improved the egg production rate and daily egg mass (*P* < 0.05), and it had a trend to increase the average daily feed intake compared with the control group (*P* = 0.053) (Table S3). The PQQ·Na_2_ supplementation also significantly improved the albumen height and Haugh unit (*P* < 0.05) and had a trend to increase the egg strength (*P* = 0.052), and no significant difference was obtained in other egg quality parameters such as eggshell thickness, eggshell color, yolk color, as well as yolk weight, eggshell weight and albumen weight (*P* > 0.05) (Table 1).

### 3.2 Egg antioxidative status and freshness during storage

**Fig. 1:**
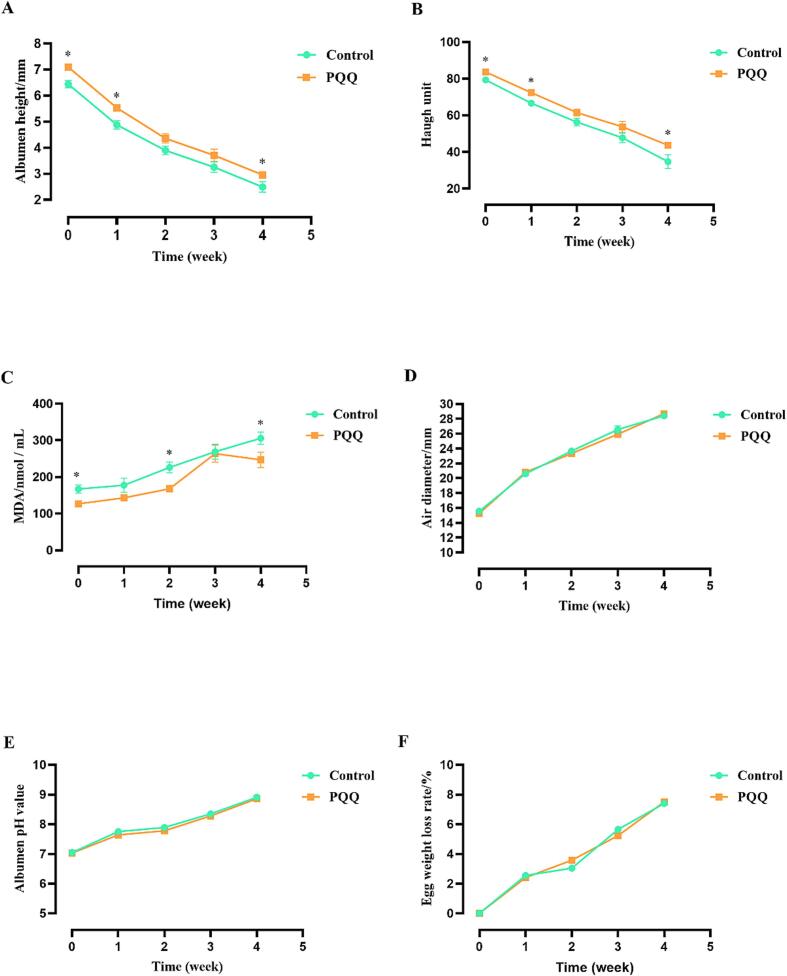
Effects of PQQ·Na_2_ on modulating the egg quality during storage. Egg albumen height (A), Egg Haught unit (B), MDA of egg yolk (C), Egg air diameter (D), Egg albumen pH value (E), and Egg weight loss rate (F). The values are shown as the means ± SD (n = 6). Broken lines that with * above are statistically distinct (*P* < 0.05).

Albumen height and Haugh unit were significantly higher (Table 1) and MDA in egg yolk was significantly lower (Table 2) in the PQQ·Na_2_ group compared to the control group when eggs were stored for 0 d (*P* < 0.05), and these variation tendencies were also observed when egg stored for 28 d (*P* < 0.05) (Fig. 1A-C). No difference was found in egg air diameter, egg albumen pH value and Egg weight loss rate (*P* > 0.05) (Fig. 1D-F).

**Table 1:** Effect of dietary PQQ·Na_2_ supplementation on egg quality of laying hens.

Items	CON	PQQ	*P*-Value
Eggshell strength, kg/cm^2^	4.28 ± 0.10	4.70 ± 0.16	0.052
Eggshell thickness, mm	0.35 ± 0.02	0.36 ± 0.01	0.521
Albumen height, mm	6.44 ± 0.14	7.09 ± 0.13	0.006
Haugh unit	79.35 ± 0.87	83.65 ± 0.78	0.004
Eggshell color	25.57 ± 0.16	24.98 ± 0.30	0.134
Yolk color	6.97 ± 0.35	6.23 ± 0.40	0.189
Yolk ratio, %	27.47 ± 0.18	27.16 ± 0.37	0.502
Shell ratio, %	10.02 ± 0.05	9.90 ± 0.14	0.444
Albumen ratio, %	62.62 ± 0.17	62.94 ± 0.46	0.608

**Table 2:** Effect of dietary PQQ·Na_2_ supplementation on egg antioxidant substances of laying hens at 8 wk.

Items	CON	PQQ	*P*-Value
Serum SOD, U/ml	239.10 ± 3.97	248.08 ± 11.15	0.476
Serum CAT, U/ml	13.17 ± 6.64	13.46 ± 4.16	0.971
Serum MDA, nmol/ml	5.17 ± 0.68	4.46 ± 0.13	0.347
Serum GSH-Px, U/ml	1912.11 ± 115.00	2280.50 ± 129.87	0.046
Liver SOD, U/mgprot	129.64 ± 11.36	137.52 ± 9.58	0.607
Liver CAT, U/mgprot	367.60 ± 49.50	316.03 ± 28.29	0.387
Liver MDA, nmol/mgprot	0.87 ± 0.03	0.72 ± 0.04	0.050
Liver GSH-Px, U/mgprot	42.13 ± 4.87	39.75 ± 3.66	0.704

### 3.3 Plasma, liver antioxidative status

Dietary PQQ·Na_2_ addition increased GSH-Px concentration in serum and decreased MDA concentration in the liver (*P* < 0.05) (Table 2). No differences in CAT, and SOD activities were obtained between the control group and the PQQ·Na_2_ treatment group (*P* > 0.05).

### 3.4 Antioxidative-related genes and proteins expression levels in liver

**Fig. 2:**
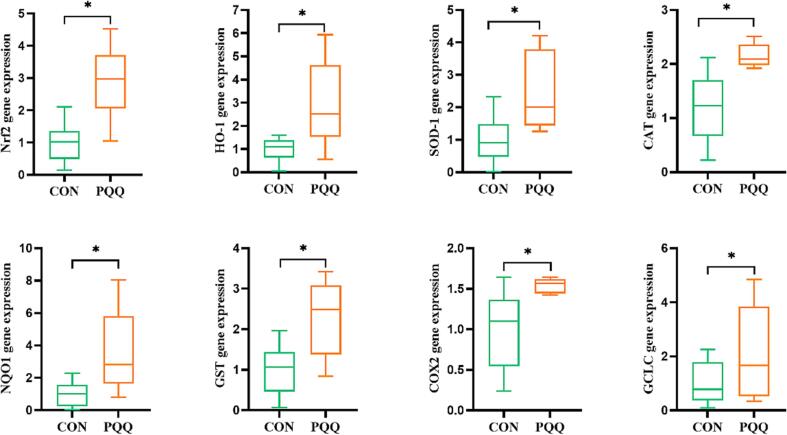
PQQ·Na_2_ increases the expression of antioxidative-related genes in liver of layers. The values are shown as the mean ± SD (n = 6). Bars that with * above are statistically distinct (*P* < 0.05).

Compared to the control treatment, PQQ·Na_2_ supplementation effectively increased the mRNA expression of Nrf2, HO-1, SOD-1, CAT, NQO1, GST, COX2 and GCLC in liver (*P* < 0.05) (Fig. 2). In like manner, the corresponding protein expression levels of Nrf2 and HO-1 were likewise higher in PQQ·Na_2_ supplementation group (*P* < 0.05) (Figure S1).

### 3.5 Microflora structure in the cecal contents

**Fig. 3:**
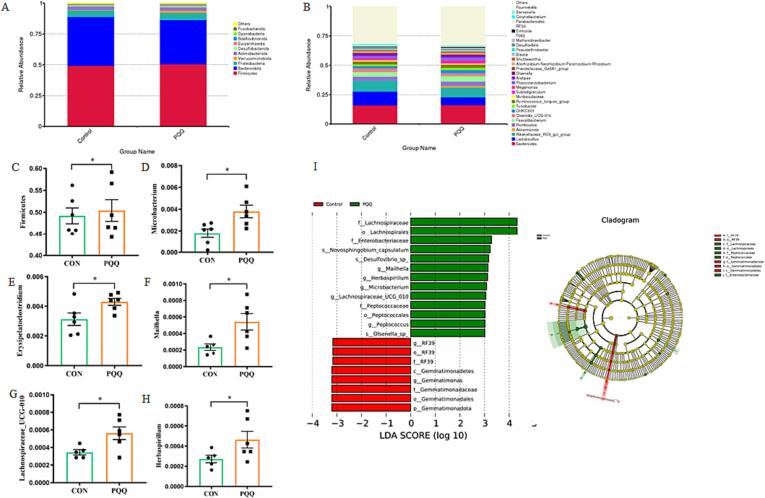
Effects of PQQ·Na_2_ on the taxon composition of gut microflora in the cecum of layers. Relative abundance of the caecal microflora of top 10 at phylum (A) and of top 30 at genus (B), relative abundance of *Firmicutes* (C), relative abundance of *Microbacterium* (D), relative abundance of *Erysipelatoclostridium* (E), relative abundance of *Mailhella* (F), relative abundance of *Lachnospiraceae_UCG-010* (G), relative abundance of *Herbaspirillum* (H), and The LEfSe analysis (I).

The influence of PQQ·Na_2_ on the gut microflora was investigated with 16S rRNA sequencing. The microbiota in the cecal content is shown in the Venn diagram (Fig. S2A). A total of 958 ASVs were shared between the two groups, with 556 unique ASVs in the control group chickens and 551 unique OTUs in the PQQ·Na_2_ supplementation group chickens. The indexes of Alpha diversity (including Chao1, Shannon and Simpson) revealed the richness and diversity of all samples, though no significant difference was observed between the two groups (Fig. S2B-D). A separation in microflora communities between the PQQ·Na_2_ supplementation and control group as indicated via the principal component analysis (PCA) and Non-Metric Multi-Dimensional Scaling (NMDS) (Fig. S2E-F), indicating the effects of PQQ·Na_2_ supplementation on microbial beta diversity. The dominant microbial composition of the cecal contents in laying hens comprised *Firmicutes*, *Bacteroidota* and *Proteobacteria* (relative abundance > 1 %) at the phylum level (Fig. 3A), with a higher relative abundance of *Firmicutes* in PQQ·Na_2_ supplementation group (*P* < 0.05) (Fig. 3B). At the genus level, we observed *Bacteroides*, *Lactobacillus*, *Rikenellaceae_RC9_gut_group*, *Romboutsia*, *Feacalibacterium, Clostridia_UCG-014*, *Ruminococcus_torques_group, Akkermansia, CHKCI001, Turicibacter, Muribaculaceae, Subdoligranulum* and *Phascolarctobacterium* were the dominant genera (relative abundance > 1 %) in two groups. A T-test was further used to analyze all the differential bacteria and we found PQQ·Na_2_ supplementation significantly increased the ratio of *Firmicutes*, *Microbacterium, Erysipelatoclostridium, Mailhella, Lachnospiraceae_UCG-010, Herbaspirillum* (*P* < 0.05) (Fig. 3C–3H), and these results were consisted with the LEfSe analysis (Fig. 3I).

## 4 Discussion

PQQ·Na_2_ has attracted substantial attention based on its ability for improving growth, development, and reproduction via several physiological and biological processes (Ikemoto, Mori, & Mukai, 2017). Especially, increasing evidence have demonstrated PQQ·Na_2_ possesses antioxidant property to regulate redox status and promote animal health (Rucker et al., 2009, Yin et al., 2019). As expected, the results collected in the present study revealed there was a significant improvement in production performance with PQQ·Na_2_ supplementation for 8 weeks. Likewise, Wang et al. (2016) noticed supplementing hens with 0.08 mg/kg PQQ·Na_2_ increased and average daily feed intake. Meanwhile, a significant increased egg mass was found in the present study, which might be related to the PQQ·Na_2_ ability of stabilize the intestinal microbiota to ensure the nutrient utilization (Zhang et al., 2020). However, Wang et al (Wang et al., 2016) found dietary inclusion of PQQ·Na_2_ did not affect the egg laying rate of laying hens during the late laying period, and they considered it was attributed to lower PQQ·Na_2_ supplied level and lower oxidation level (Zhang et al., 2010), suggesting PQQ·Na_2_ can be more effective as physiological status of layers are challenged (Wang et al., 2020). Moreover, the increase of albumen height and Haugh unit of fresh eggs and stored eggs, as well as egg eggshell strength were observed in this study, which was in accordance with Sun et al. (Sun et al., 2014), suggesting that dietary PQQ·Na_2_ supplementation advanced the internal egg quality. The improvement of internal egg quality may be associated with the increase of the antioxidative properties of layers (Xie et al., 2019). As there are various stressors in commercial layers with intensive feeding systems that result in redox imbalance, decreased reproductive system function as well as decreased protein synthesis and transport capacity (Mishra & Jha, 2019), with the display of declined albumen height and Haugh unit of fresh eggs and greatly shortening the shelf life of eggs (Silversides & Scott, 2001).

The organism’s redox state has been in a dynamic equilibrium, and oxidative stress, disease, and aging would arise in case of antioxidant defense function is impaired (Yu et al., 2015). Endogenous antioxidant members within the enzymatic antioxidant system like GSH-Px, SOD, and CAT, play significant roles in preserving the organism from oxidative stress (Wang et al., 2020). MDA, the proverbial product of lipid peroxidation, is an important biomarker of oxidant status. As an effective antioxidant, PQQ·Na_2_ could restore the activities of GSH-Px and SOD to reverse the oxidant stress of layers induced by oxidized oil (Wang et al., 2016). Additionally, it was confirmed that supplementation of PQQ·Na_2_ exhibited protective effects on oxidant stress of broilers facing LPS challenge not only by operation of antioxidant enzymes like GSH-Px, SOD, CAT, but also by neutralization of lipid peroxidation product MDA (Zheng et al., 2020). Furthermore, in vivo and in vitro studies also demonstrated treatment with PQQ·Na_2_ increased the concentration of SOD, and decreased MDA concentration in weaned pigs (Huang et al., 2021). Similarly, in the current study, the enhanced GSH-Px activity and mRNA expression of antioxidant enzymes or the decreased activity of MDA might be related to the dietary supplementary of PQQ·Na_2_ appearing to help maintain redox status in the high metabolism of laying hens (Wang et al., 2015). Intriguingly, we also found dietary PQQ·Na_2_ decreased the MDA content in fresh egg yolk as well as stored egg yolk, which were in accordance with a previous finding that MDA contents of eggs were decreased with the natural antioxidant supplementary (Zhou et al., 2021). This study was the first to report the antioxidative capacity improvement of the eggs by PQQ·Na_2,_ but the reason is not clear, and it might be inferred PQQ·Na_2_ increased the transshipment and accumulation of hydrophobic amino acids in eggs to increase the peptides solubility in lipids and promote approachability to radical species (Wang et al., 2020). Nevertheless, more and further investigations are still needed to illustrate the underlying mechanism for this result.

Protection of the body from damage produced by oxidative stress is often associated with the activation of antioxidant signaling pathways (Huang et al., 2021), while the Nrf2 pathway has been considered as a primary modulation of cell rebellion to oxidative injury (Liu et al., 2021). Previous studies have demonstrated that the Nrf2 pathway participated in protective effects of PQQ·Na_2_ on attenuating stimulated oxidative stress by increasing the expression levels of Nrf2 and HO-1 in weaned pigs (Huang et al., 2021). As a redox transcription factor, Nrf2 occupies a crucial part in effectively restoring oxidative status (Qin & Hou, 2016). When suffering from oxidative stress, Nrf2 will be phosphorylated and then translocated to the nucleus to up-regulate the expression of HO-1 (Guo et al., 2017), and activated a set of metabolizing enzymes such as NQO1 (Zhang et al., 2008) and GST (Szklarz, 2013), and other downstream genes. HO-1 is a stress protein that protects tissue and cellular from various injury (Zhao et al., 2019), and up-regulated HO-1 can ameliorate oxidative damage by accommodating the levels of MDA, SOD, and GSH-Px (Wang et al., 2019). Expectedly, in the current study, dietary PQQ·Na_2_ boosted the liver Nrf2 and HO-1 mRNA expression and protein expression, combined with the downstream genes for example NQO1, SOD-1, CAT, GST, indicating PQQ·Na_2_ activated Nrf2/HO-1 pathway and regulated its downstream genes.

The gut microbiota plays an indispensable role to keep the host healthy by performing immunologic, metabolic and protective functions (Zhang et al., 2022), and the contribution of gut microbiota to the antioxidant capacity of the host has attracted considerable attention (Chen et al., 2022). Whereas, until now, limited data about the effect of PQQ·Na_2_ on the microbiota of layers have been published, much less on the relationship between gut microbiota and antioxidant capacity of the host, though, a previous study supported PQQ could develop a program of microbial dysbiosis in obese mice with liver fibrosis (Friedman et al., 2018). Not exactly consistent with the previous study about dietary PQQ·Na_2_ in sows increased the *Firmicutes* level and decreased the *Escherichia* abundance to improve the antioxidant status (Wang et al., 2020), in the present study, PQQ·Na_2_ administration obtained a remarkable increase in some beneficial bacteria such as *Firmicutes*, *Lachnospiraceae*, *Herbaspirillum* and so on. Of which, *Lachnospiraceae*, belonging to the *Firmicutes*, is identified as one of the dominant bacteria and performed a part in sustaining host health (Biddle et al., 2013). It was proved metabolites produced by gut microbiota were connected with physiological functions inside and outside the gut (Fan & Pedersen, 2021). Indeed, a recent study found the increased abundance of *Lachnospiraceae* families could enhance the liver antioxidant capacity of mice to suppress oxidative stress which was attributed to its high capacity of producing a novel antioxidant called cysteine persulfide (CysSSH) (Uchiyama et al., 2022). Moreover, *Lachnospiraceae* were reported as beneficial microbiota and promote butyrate-producing (Li et al., 2019), which were known to provide a source of energy, to improve antioxidant status and immunity (Zhang & Piao, 2022). And butyrate was found attenuate oxidative stress and neuron apoptosis in mice with activation of Nrf2 pathway (Li et al., 2021). Here we suspected that PQQ·Na_2_ supplementary increased the relative abundance of *Lachnospiraceae* to promote the antioxidant capacity of layers, yet the particular connection between PQQ·Na_2_ antioxidant effect and gut microbiota in the current study is still unclear. Therefore, it will be of interest to identify the product from gut microbiota and investigate its potential protective function mechanisms of improving host antioxidant capacity.

## 5 Conclusion

**Fig. 4:**
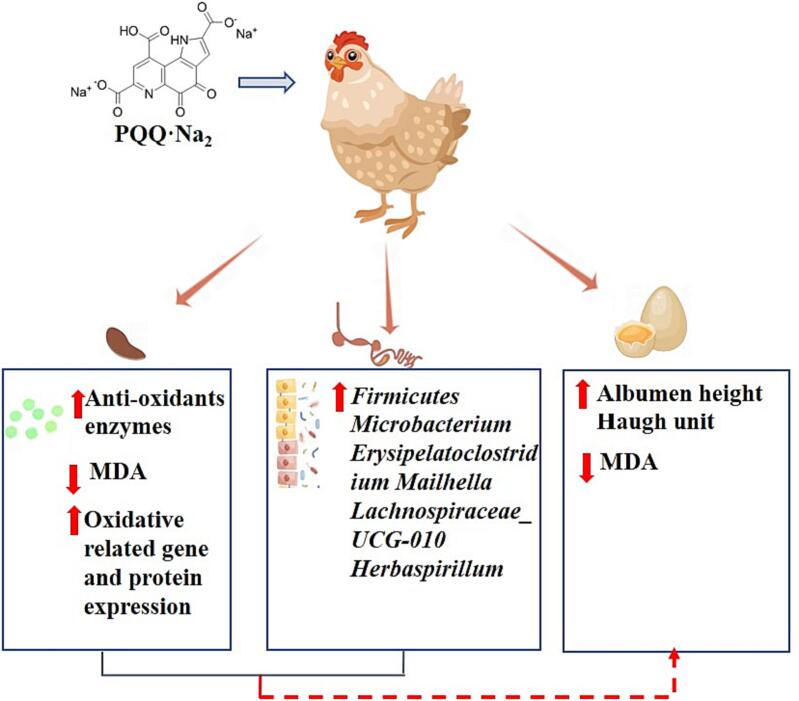
The overview of the effect of PQQ·Na_2_ supplementation in layers and eggs.

In summary, the results in the present study indicated dietary supplementary of PQQ·Na_2_ increased the laying performance of layers, antioxidant capacity of layers and eggs as appeared in up-regulated antioxidant system of layers and decreased concentration of MDA in eggs. And this observation might attribute to a positive activation of the Nrf2/HO-1 pathway as well as un-regulated beneficial gut microbiota like *Firmicutes*, *Microbacterium*, *Erysipelatoclostridium*, *Mailhella*, *Lachnospiraceae_UCG-010*, and *Herbaspirillum* (Fig. 4). Our findings laid a foundation for further study of the relationship among PQQ·Na_2,_ gut microbiota and antioxidant capacity_._

## CRediT authorship contribution statement

**Dan Shao:** Conceptualization, Investigation, Formal analysis, Data curation, Writing – original draft. **Liangji Liu:** Investigation, Software, Resources. **Haibng Tong:** Methodology, Data curation, Writing – review & editing. **Shourong Shi:** Conceptualization, Resources, Supervision, Project administration, Writing – review & editing.

## Declaration of competing interest

The authors declare that they have no known competing financial interests or personal relationships that could have appeared to influence the work reported in this paper.

## Data Availability

The 16S rRNA sequence data were submitted to the National Center for Biotechnology Information (NCBI). The bioproject number for 16S sequencing data is PRJNA915744.
