# Supplementary material for: Dietary pyrroloquinoline quinone improvement of the antioxidant capacity of laying hens and eggs are linked to the alteration of Nrf2/HO-1 pathway and gut microbiota
Source: Food Chem X. 2023 Nov 30;20:101021. doi: 10.1016/j.fochx.2023.101021 (PMC10740097; doi:10.1016/j.fochx.2023.101021)
Supplement: Supplementary data 1 [file mmc1.docx]

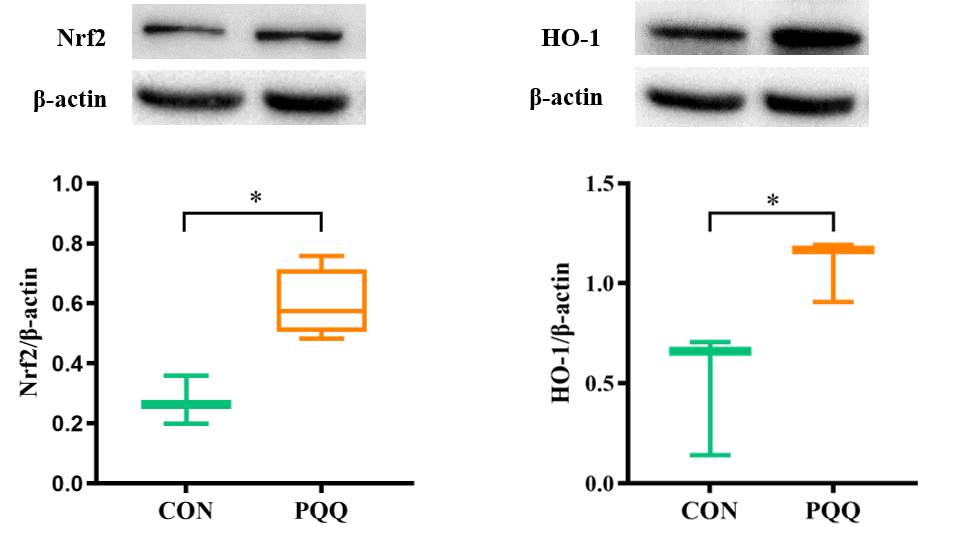


Fig.S1 PQQ·Na_2_ increases the protein expression of Nrf2 and HO-1. The values are shown as the mean ± SD (n = 6). Bars that with * above are statistically distinct (*P*<0.05).


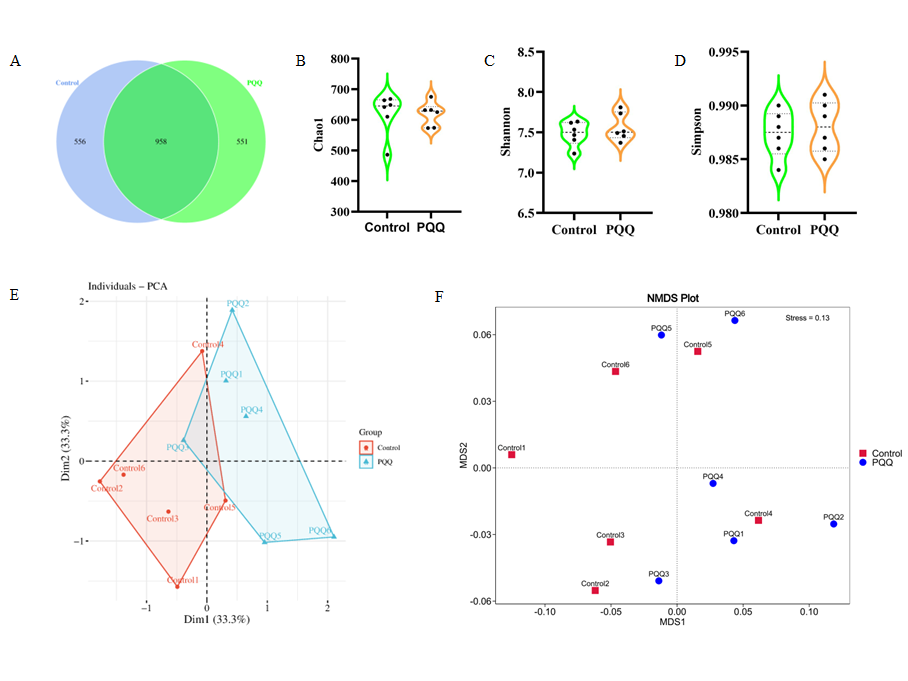


Fig.S2 Effects of PQQ·Na_2_ on the structure and diversity of gut microflora in the cecum of layers. The Venn diagram of the numbers of shared and unique ASVs in the gut microflora (A), Chao 1 index (B), Shannon index (C), and Simpson index (D) of species diversity within and between two groups, PCA = principal co-ordinates analysis (E) and Non-metric multi-dimensional NMDS analysis (F) of the overall structure community of the gut microbiota.
