# Supplementary material for: Dietary pyrroloquinoline quinone improvement of the antioxidant capacity of laying hens and eggs are linked to the alteration of Nrf2/HO-1 pathway and gut microbiota
Source: Food Chem X. 2023 Nov 30;20:101021. doi: 10.1016/j.fochx.2023.101021 (PMC10740097; doi:10.1016/j.fochx.2023.101021)
Supplement: Supplementary data 2 [file mmc2.docx]

**Table S1** Composition and nutrient levels of the basal diet (air-dry basis)

| Items | Content (%) |
| --- | --- |
| Corn, ground | 60.00 |
| 43% Soybean meal | 28.43 |
| Soybean oil | 1.00 |
| Limestone | 8.30 |
| Dicalcium Phosphate | 1.50 |
| DL-methionine | 0.15 |
| Salt | 0.30 |
| 70% Choline chloride | 0.09 |
| Mineral premix^1^ | 0.20 |
| Vitamin premix^2^ | 0.03 |
| Total | 100 |
| Nutrient levels |  |
| Metabolisable energy (MJ kg^-1^) | 11.34 |
| Crude protein | 17.05 |
| Calcium | 3.52 |
| Available P | 0.44 |
| Lysine | 0.83 |
| Methionine | 0.41 |
| Cystine | 0.24 |

Note: Superscript ^1^ Mineral premix provided the following per kg of the diet: copper sulfate, 20 mg; iron sulfate, 144 mg; zinc oxide, 144 mg; manganese sulfate, 120 mg; iodine, 0.96 mg, selenium 0.7 mg; cobalt, 1 mg.

Superscript ^2^ Vitamin premix provided the following per kg of the diet: Vitamin A, 13 500 IU; Vitamin D3, 3 900 IU; Vitamin E, 30 IU; Vitamin K3, 4.8 mg; Vitamin B1, 3 mg; Vitamin B2, 7.5 mg; Vitamin B6, 6 mg; Vitamin B12, 0.024 mg; folic acid, 1.5 mg; biotin, 0.18 mg; niacin, 45 mg; calcium pantothenate, 18 mg.

**Table S2** Gene-specific primers for real-time quantitative reverse transcription PCR

| Genes | Primers (5’-3’) | Gene number | Product size, bp |
| --- | --- | --- | --- |
| Nrf2 | Forward: GGTGACACAGGAACAACA | NM_205117.2 | 223 |
|  | Reverse: AAGTCTTATCTCCACAGGTAG |  |  |
| HO-1 | Forward: CTGAAGGAAGCCACCAAG | NM_205344.2 | 136 |
|  | Reverse: CCAGAGCAGAGTAGATGAAG |  |  |
| SOD-1 | Forward: GCTTGTGGTGTAATTGGAAT | NM_205064.2 | 159 |
|  | Reverse: AGACAGCAGAGTAGTAATGAG |  |  |
| CAT | Forward: CACTGTTGCTGGAGAATCT | NM_001031215.2 | 161 |
|  | Reverse: GGCTATGGATGAAGGATGG |  |  |
| NQO1 | Forward: CACCATCTCTGACCTCTAC | NM_001277620.2 | 173 |
|  | Reverse: CCGCTTCAATCTTCTTCTG |  |  |
| GST | Forward: CCATCCTCAACTACATAGCA | NM_001001777.2 | 91 |
|  | Reverse: GCCAGTCCTTCCACATAC |  |  |
| GCLC | Forward: AGGCTATGTGTCCGATATTG | XM_419910.7 | 100 |
|  | Reverse: GTTGTTCTTCAGTGGCTCTA |  |  |
| β-Actin | Forward: ATGTGGATCAGCAAGCAGGAGTA | NM_205518.2 | 127 |
|  | Reverse: TTTATGCGCATTTATGGGTTTTGT |  |  |

**Table S3** Effect of dietary PQQ·Na_2_ supplementation on production performance of laying hens

| Items | CON | PQQ | *P*-Value |
| --- | --- | --- | --- |
| Egg production, % | 94.02±1.50 | 97.52±0.30 | 0.046 |
| Average daily feed intake, g | 119.42±0.50 | 120.99±1.26 | 0.053 |
| Average egg weight, g | 59.44±0.42 | 59.78±0.43 | 0.576 |
| Daily egg mass g/d | 55.88±0.91 | 58.30±0.40 | 0.036 |
| FCR | 2.14±0.04 | 2.08±0.01 | 0.173 |
